# High-Level Heterologous Expression of Endo-1,4-β-Xylanase from *Penicillium citrinum* in *Pichia pastoris* X-33 Directed through Codon Optimization and Optimized Expression

**DOI:** 10.3390/molecules24193515

**Published:** 2019-09-27

**Authors:** Chanika Ouephanit, Nassapat Boonvitthya, Sophie Bozonnet, Warawut Chulalaksananukul

**Affiliations:** 1 Program in Biotechnology, Faculty of Science, Chulalongkorn University, Bangkok 10330, Thailand; chanika.gae@gmail.com; 2 Department of Botany, Faculty of Science, Chulalongkorn University, Bangkok 10330, Thailand; 3 Biofuels by Biocatalysts Research Unit, Department of Botany, Faculty of Science, Chulalongkorn University, Bangkok 10330, Thailand; 4 Innovation Institute, PTT Public Co., Ltd., Ayutthaya 13170, Thailand; nassapat.b@pttplc.com; 5 LISBP, Université de Toulouse, CNRS, INRA, INSA, 31077 Toulouse, France; sophie.bozonnet@insa-toulouse.fr

**Keywords:** codon optimization, overexpression, *Penicillium citrinum*, *Pichia pastoris*, *P_GAP_*, *P_AOX1_*, xylanase A

## Abstract

Most common industrial xylanases are produced from filamentous fungi. In this study, the codon-optimized *xynA* gene encoding xylanase A from the fungus *Penicilium citrinum* was successfully synthesized and expressed in the yeast *Pichia pastoris*. The levels of secreted enzyme activity under the control of glyceraldehyde-3-phosphate dehydrogenase (*P_GAP_*) and alcohol oxidase 1 (*P_AOX1_*) promoters were compared. The Pc Xyn11A was produced as a soluble protein and the total xylanase activity under the control of *P_GAP_* and *P_AOX1_* was 34- and 193-fold, respectively, higher than that produced by the native strain of *P. citrinum.* The Pc Xyn11A produced under the control of the *P_AOX1_* reached a maximum activity of 676 U/mL when induced with 1% (*v/v*) methanol every 24 h for 5 days. The xylanase was purified by ion exchange chromatography and then characterized. The enzyme was optimally active at 55 °C and pH 5.0 but stable over a broad pH range (3.0–9.0), retaining more than 80% of the original activity after 24 h or after pre-incubation at 40 °C for 1 h. With birchwood xylan as a substrate, Pc Xyn11A showed a *K*_m(app)_ of 2.8 mg/mL, and a *k*_cat_ of 243 s^−1^. The high level of secretion of Pc Xyn11A and its stability over a wide range of pH and moderate temperatures could make it useful for a variety of biotechnological applications.

## 1. Introduction

Plant cell walls are the main polysaccharide-containing renewable resource on earth, and are generated by photosynthesis [1]. Hemicellulose, a group of heteropolysaccharides, is the main polysaccharide component of plant cell walls, and in most plants, xylan (β-1,4-linked xylose residues with side branches of α-glucuronic acid and α-arabinofuranose) forms the major component and accounts for approximately one third of all renewable organic carbon on earth [2]. As xylan is a complex heteropolysaccharide, the complete conversion of the xylan requires the interaction of several main-chain- and side-chain-cleaving enzymes. Of these, xylanases (endo-1,4-β-xylanase, EC 3.2.1.8) play a key role in xylan hydrolysis to xylooligosaccharides, which in turn can be converted to xylose by β-xylosidases [3].

Xylanases are widely used in various biotechnological applications. Examples include serving as bio-bleaching agents in the pulp and paper industry [4], improving digestibility and enhancing the efficiency of nutrient utilization in animal feed [5,6], increasing dough volume in the bakery industry [7], clarification of juices [8], improving the filtration rate and reduction of viscosity in the brewing industry [9], and releasing xylose that can be fermented to value products, such as biofuels and xylitol [10,11].

Xylanases are widespread in nature and have been reported in many microorganisms [2,12,13,14,15]. Most commercial xylanases are mainly produced by *Trichoderma* spp. and *Aspergillus* spp [16]. However, a number of xylanase-producing fungi have also been isolated from various sources, including soil, decaying woods, agricultural waste materials, and mangrove forests [17,18,19,20]. *Penicillium citrinum* appears to be an interesting xylanase-producing fungus since its xylanases are stable over a wide pH range [21], but there have been few other reports of xylanase from *P. citrinum* [22].

Heterologous gene expression by the methylotrophic yeast *Pichia pastoris* is a useful alternative for producing genetically engineered enzymes for research as well as industrial purposes [23]. It is considered to be non-toxigenic and non-pathogenic, and several processes based on this organism have been classified as generally recognized as safe (GRAS) by the Food and Drug Administration (FDA, White Oak, MD, USA) [24]. Moreover, *P. pastoris*, as an excellent host for the production of recombinant proteins, provides additional benefits, such as a rapid growth rate to high cell density in inexpensive and non-complex culture medium, and ease of purification of heterologously-expressed recombinant proteins [25,26].

Codon optimization technology is a commercialized algorithm for heterologous gene expression. The DNA sequence used to encode a protein varies between different organisms. To obtain a high expression level in the new host, changing the foreign DNA sequence into that which matches the host requirements for expression is frequently required [27,28].

In this study, the *xynA* gene encoding a GH family-11 xylanase A from the fungus *P. citrinum* (Pc Xyn11A) strain FERM P-15944 was synthesized by optimization of codon usage and then cloning in the heterologous yeast host *P. pastoris*. A comparison of the expression level from a constitutive and inducible expression system, under the control of glyceraldehyde-3-phosphate dehydrogenase (*P_GAP_*) and alcohol oxidase 1 (*P_AOX1_*) promoters, respectively, was performed in order to improve the production and to characterize the Pc Xyn11A enzyme. This is the first report on the expression of an optimized codon *xynA* gene from *P. citrinum* in *P. pastoris*.

## 2. Results and Discussion

### 2.1. Construction of Expression Vectors, Transformation and Selection

**Figure 1:**
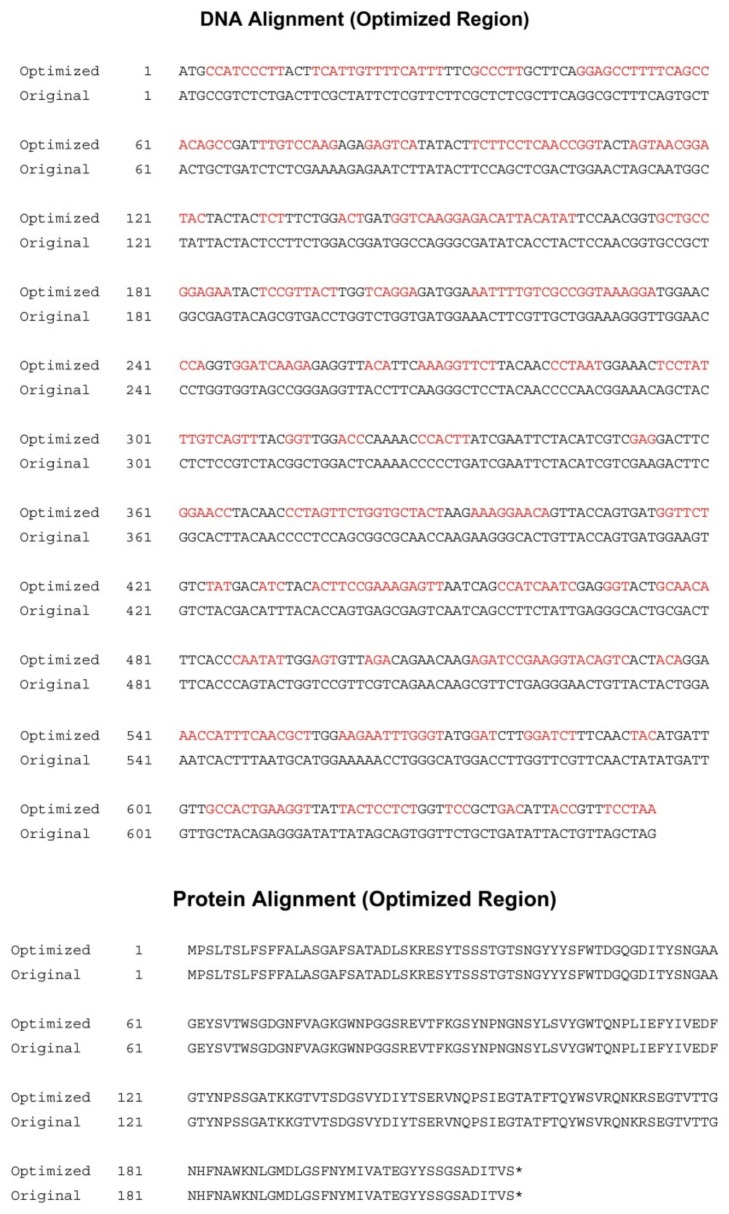
DNA alignment and protein alignment of the original and codon-optimized (for expression in *P. pastoris*) *xynA* gene from *P. citrinum* FERM P-15944.

The *xynA* gene from *P. citrinum* FERM P-15944 was codon optimized to enhance the translation efficiency, and was then expressed in *P. pastoris* X-33 to improve the expression level and study xylanase A. The mature protein-coding *xynA* gene consists of 573 nucleotides and encodes a protein of 190 amino acid residues. The optimized DNA sequence, aligned with the native gene, and the protein alignment, are shown in Figure 1, where due to the codon bias of *P. pastoris*, 138 codons were changed. However, the alignment clearly showed that both sequences encoded a protein with the same amino acid sequences. The codon usage bias was changed by upgrading the codon adaptation index (CAI) from 0.69 to 0.83, giving a smoother GC content curve and eliminating the negative cis-acting elements. The potential *N*- and *O*-glycosylation sites of Pc Xyn11A were predicted using the NetNGlyc 1.0 Server (http://www.cbs.dtu.dk/services/NetNGlyc/) and the NetOGlyc 4.0 Server (http://www.cbs.dtu.dk/services/NetOGlyc-4.0/), respectively. The results showed that there was no *N*-glycosylation, whilst there were four possible *O*-glycosylation sites.

Several studies have reported that heterologous protein production by GAP is better than AOX1system [29,30,31,32], on the other hand, others have reported that AOX1system is better for protein production [33,34,35], and so both were examined for the expression of Pc Xyn11A in *P. pastoris* in this study. Both the pGAPZα A and pPICZα A plasmids contain the native *Saccharomyces cerevisiae* α-factor secretion signal for extracellular production of protein and the Zeocin resistance gene for positive selection in *E. coli* and *P. pastoris*. The gene encoding the mature Pc Xyn11A was cloned in frame with the α-factor secretion signal under the control of *P_GAP_* or *P_AOX1_* to produce the recombinant expression plasmids pGAPZαA-xynA and pPICZαA-xynA, respectively. After transformation into *P. pastoris* X-33, the transformants were selected on a YPDS agar plate containing 100–2000 µg/mL Zeocin.

For pGAPZαA-xynA, YPDS agar plates containing 100 µg/mL Zeocin showed the highest number of transformants, and this number decreased as the concentration of Zeocin increased, with no colonies growing at 2000 µg/mL Zeocin. As *P_GAP_* is a constitutively expressed promoter, the transformants were preliminary selected on YP with 0.2% Azo-xylan agar plates, where all transformants produced xylanase activity, as determined by the clear halo indicating that the substrate was hydrolysed and released soluble dye-labelled fragments around the colony. The negative control, untransformed *P. pastoris* X-33, did not show any clear zone. For pPICZαA-xynA, the transformants grew on all YPDS agar plates containing 100–2000 µg/mL Zeocin, although the number of transformants decreased when the concentration of Zeocin was increased.

### 2.2. Expression of xynA-PC in P. pastoris

All transformants were cultured and monitored every 24 h for growth and xylanase activity in the supernatant. The wild type *P. pastoris* X-33 had no detectable extracellular xylanase activity. For the constitutive expression (*P_GAP_*), the growth of all transformants reached a steady state after 48 h, whereas for the methanol-inducible expression (*P_AOX1_*), all transformants were still in the growth phase after 5 days with induction with methanol every 24 h. The maximum xylanase activity of the pGAPZαA-xynA transformants (Pp-gap-xynA) was from a clone picked on a YPDS agar plate containing 100 µg/mL Zeocin. After 48 h of culture, the xylanase activity reached 119.5 µmol/mL/min, which was 34-fold higher than that of the native strain *P. citrinum* at 72 h [21].

For pPICZαA-xynA-containing clones, in order to maximize the Pc Xyn11A production level, the methanol concentration was varied in BMMY medium during induction. The maximum xylanase activity (676 µmol/mL/min) of the pPICZαA-xynA transformants was obtained from a clone selected on a YPDS agar plate containing 500 µg/mL Zeocin and cultured with 1% (*v/v*) methanol in BMMY and induced with 1% (*v/v*) methanol every 24 h for 5 days, an activity that was 193-fold higher than that of the native fungus *P. citrinum* [21].

Besides the synthesis of Pc Xyn11A in *P. pastoris* X-33 in this study, the secretion of xylanase in *P. pastoris* strain GS115 using the integrative yeast expression vector pPIC3.5 has been reported previously [21]. However, the obtained xylanase activity was 7- and 39.7-fold lower than that for the Pc Xyn11A obtained in this study under the *P_GAP_* and *P_AOX1_*, respectively. Ohta et al. [36] tried to improve the xynA protein secretion in *E. coli* BL21(DE3) by fusion with the signal peptide of *Aureobasidium pullulans XynI* in place of the native one of *xynA* from *P. citrinum*. However, the obtained xylanase activity from this heterologous construct in *E. coli* was 7.1- and 40.2-fold lower than that of Pc Xyn11A under the *P_GAP_* and *P_AOX1_*, respectively, in this study. The *xynA* from *P. citrinum* has also been investigated in *Yarrowia lipolytica* to enhance the xynA protein production by fusion of the preproLIP2 secretion signals compared to the native one of *xynA* from *P. citrinum* [37]. Nevertheless, the obtained xylanase activity from Pc Xyn11A under the *P_AOX1_* reported in this study was 17.7- and 3.7-fold higher than that of the recombinant XynA using native and preproLIP2 secretion signals, respectively (Table 1).

### 2.3. Purification, SDS-PAGE and Zymography Analysis

**Figure 2:**
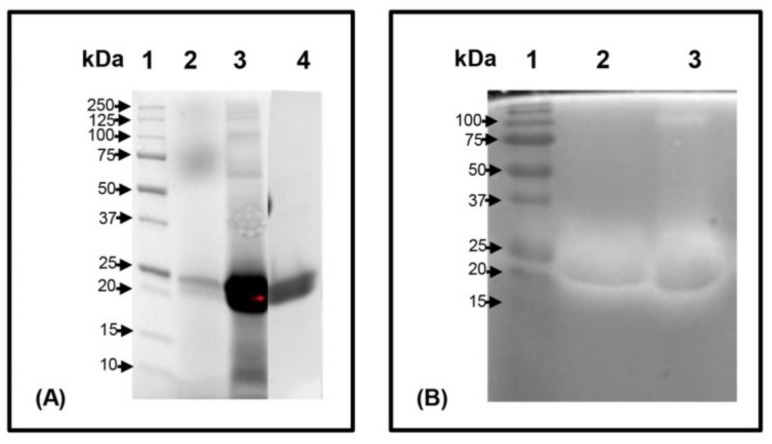
SDS-PAGE analysis and zymography of Pc Xyn11A produced in *P. pastoris* X-33. (**A**: SDS-PAGE), (**B**: Zymogram of xylanase). Lane 1: Molecular weights of the markers, Lane 2: culture supernatant, Lane 3: ultrafiltration (10 kDa MWCO) concentrated Pc Xyn11A, Lane 4: Q Sepharose anion exchange chromatography purified Pc Xyn11A.

The secreted Pc Xyn11A produced by the pPICZαA-xynA transformant was chosen for purification and characterization. The purification of Pc Xyn11A is summarized in Table 2. After ultrafiltration, the concentrate still retained 95.3% of the initial activity, but this then dramatically decreased to 26.4% after anion exchange chromatography. Nevertheless, this protocol afforded a 34-fold purification of the Pc Xyn11A enzyme from the culture supernatant and led to purification to apparent homogeneity with a final specific activity of 1259 U/mg. The molecular weight of the Pc Xyn11A enzyme was estimated by running the standard SDS-PAGE (without Azo-xylan) and was confirmed by running the zymogram (containing 1% Azo-xylan) at the same time. It was estimated to be 20 kDa, the same as that of the native fungal *P. citrinum* xylanase. Moreover, these results suggest that the Pc Xyn11A did not undergo post-translational glycosylation. However, post-translational glycosylation has previously been observed during recombinant expression of the *xynA* in *Y. lipolytica*, which required deglycosylation before purification [37]. The Pc Xyn11A in this study represented the major protein in the culture supernatant, and was resolved as a single band after anion exchange chromatography (Figure 2). These results support previous reports that *P. pastoris* secretes only very low levels of native proteins and the secreted heterologous protein constitutes the majority of the total protein in the medium [38,39], making it easier to recover the foreign secreted protein from the culture supernatant.

### 2.4. Effect of pH and Temperature

**Figure 3:**
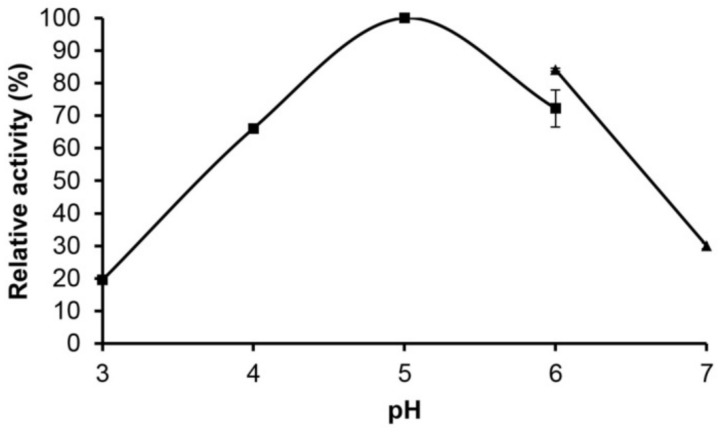
Effects of pH on the activity of purified Pc Xyn11A over a pH range of 3.0 to 7.0 under a standard enzyme assay condition. The buffers used were 60 mM citric acid–sodium citrate buffer (pH 3.0–6.0) (solid square) and 60 mM sodium phosphate buffer (pH 6.0–7.0) (solid triangle).

**Figure 4:**
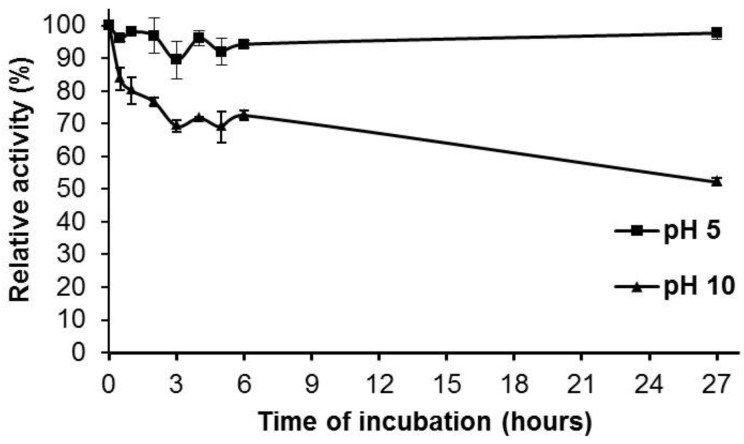
Stability of the purified Pc Xyn11A at pH 5.0 and 10.0 at room temperature measured under a standard enzyme assay condition.

With respect to the pH, the purified Pc Xyn11A enzyme showed an optimum activity at pH 5.0 (Figure 3), and a pH stability over a pH range of 3.0–9.0. It retained more than 80% of the original activity after 24 h at these pH values, but at pH 10.0, it decreased moderately to 70% and 50% after 2 and 27 h, respectively (Figure 4).

**Figure 5:**
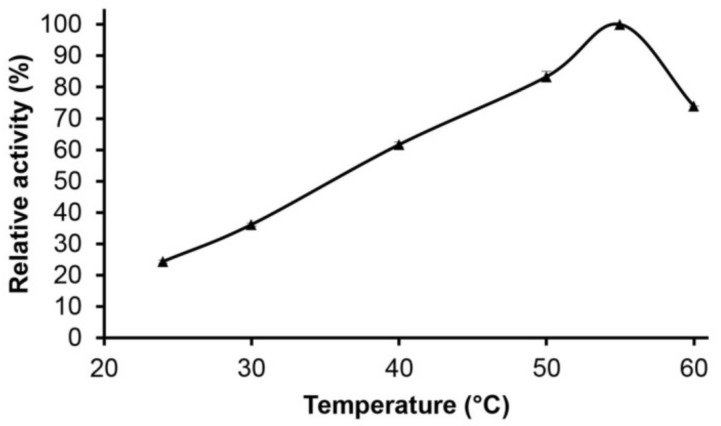
Effect of temperature on activity of purified Pc Xyn11A under a standard enzyme assay condition.

**Figure 6:**
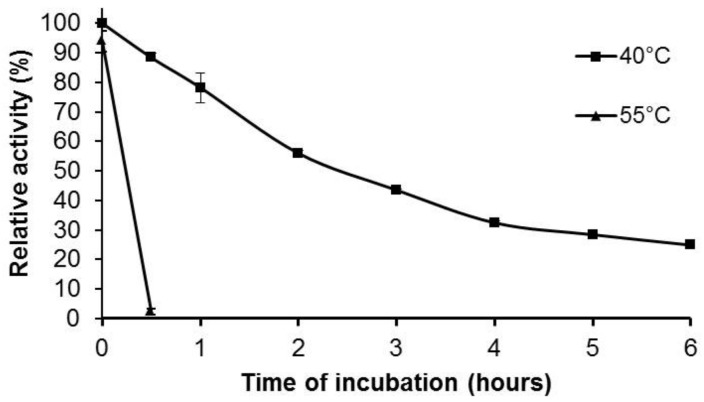
Thermal stability of purified Pc Xyn11A at 40 and 55 °C measured under a standard enzyme assay condition.

For the effect of temperature on the enzymatic activity and thermostability of the purified Pc Xyn11A, the xylanase activity at pH 5.0 increased from about 60% maximum at 40 °C to the maximum (100%) at 55 °C, designated as the optimum temperature for the enzyme activity, and then decreased to about 70% activity at 60 °C (Figure 5). The enzyme remained stable up to 40 °C, retaining around 80% of the original activity after a 1 h incubation, and then gradually declined to 20% after 6 h (Figure 6).

Endoxylanases previously reported from fungal sources usually show an optimum pH in the range of 4.0–7.0 and an optimum temperature between 40 °C and 60 °C [40,41]. The Pc Xyn11A in this study showed a similar temperature and pH optima to the xylanases from the native strain *P. citrinum* [21], *Aspergillus awamori* VTCC-F312 [42] and *P. citrinum* produced in *Y. lipolytica* [37]. However, the pH stability of Pc Xyn11A in this study at pH 10.0 retained around 50% of the original activity after a 27 h incubation, which was more activity than when heterologously expressed in *Y. lipolytica* at pH 10.0 (30% after 24 h). This could be due to the post-translational modifications. Glycosylation is one of the most important post-translational modifications in eukaryotic cells, with significant effects on protein folding, conformation, activity and stability. The modification of proteins through enzymatic glycosylation is controlled by factors that differ greatly among cell types and species [43,44]. Moreover, the pH stability of Pc Xyn11A in this study was much broader than those reported from *A. awamori* VTCC-F312 (pH 4.0–8.0) [42], *Achaetomium* sp. Xz-8 (pH 5.0–10.0) [9], *Aspergillus fumigatus* MKU1 (pH 4.0–8.0) [45], *Bacillus pumilus* ARA (pH 5.8–8.2) [46], *Bacillus* sp. GA1(6) (pH 4.0–7.0) [47] and *Penicillium oxalicum* (pH 3.0–5.0) [48]. These results show that the purified Pc Xyn11A enzyme of this study was stable for a long time over a wide pH range, and so could potentially be applied to many industries. For example, in the bakery industry, xylanase produced from *Aspergillus niger* was used to improve dough characteristics and bread quality [49]. It could be especially useful for industries that need xylanase that is stable over a wide pH range, such as clarification of fruit juice [8].

### 2.5. Kinetic Parameters

The Pc Xyn11A kinetics were evaluated at 40 °C and a pH of 5.0 using nine different birchwood xylan (substrate) concentrations. The *K*_m(app)_, *V*_max_ and *k*_cat_ were calculated to be 2.8 mg/mL, 310.7 µmol/min and 243 s^−1^, respectively. The Pc Xyn11A *k*_cat_ was higher than that previously reported for some other xylanases, such as those from *Malbranchea cinnamomea* and *Bacillus cellulosilyticus* with *k*_cat_ values of 4.26 and 165.8 s^−1^, respectively [50,51]. In comparison to other family 11 β-1,4 xylanases, the *K*_m(app)_ of the Pc Xyn11A of this study was lower than that of the native *Aspergillus niger* xylanase (Native XylA), recombinant *A. niger* xylanase produced in *Pichia pastoris* (reXylA), *Nonomuraea flexuosa* xylanase produced in *Trichoderma reesei* (Nf Xyn11A) and *P. citrinum* xylanase produced in *Y. lipolytica* (re-xynA) [37,52,53], as shown in Table 3. The difference in *K*_m(app)_ of Pc Xyn11A expressed in *P. pastoris* and *Y. lipolytica* could be due to the difference in glycosylation, as glycans can alter the substrate recognition, specificity and binding affinity, and turnover rates [54]. The lower *K*_m(app)_ of Pc Xyn11A in this study indicated that it had a higher affinity for birchwood xylan. The Pc Xyn11A also had a higher turnover number (*k*_cat_) and catalytic efficiency (*k*_cat_/*K*_m(app)_), indicating that it has higher catalytic activity.

## 3. Materials and Methods

### 3.1. Gene, Plasmids, Strains, Culture Conditions and Materials

The *xynA* gene from *P. citrinum* FERM P-15944 was synthesized with codon optimization for expression in *P. pastoris* based on the nucleotide database (GenBank: accession no. AB198065.1) [21] and introduced into the pUC57-Kan vector by GenScript (Piscataway, NJ, USA). The *P. pastoris* expression kit, including the *P. pastoris* strain X-33, constitutive expression vector (pGAPZα A), methanol-inducible expression vector (pPICZα A) and Zeocin were all from Invitrogen (Carlsbad, CA, USA). All restriction enzymes and Phusion High-Fidelity DNA polymerase were from New England BioLabs (Beverly, MA, USA). Birchwood xylan was from Sigma-Aldrich (St Louis, MO, USA), Azo-xylan was from Megazyme (Wicklow, Ireland).

*Pichia pastoris* was cultured in YPD medium 1% (*w/v*) yeast extract, 2% (*w/v*) bacteriological peptone and 2% (*w/v*) dextrose at 30 °C with shaking at 200 rpm. The transformants were selected on YPDS plates (YPD with 1 M sorbitol) containing 100–2000 µg/mL Zeocin. The pGAPZα A transformants were chosen for preliminary selection on YP (YPD without dextrose) with 0.2% (*w/v*) Azo-xylan.

*Escherichia coli* DH5α (Gibco) was used for vector propagation and was grown in a Luria-Bertani (LB) medium at 37 °C with either kanamycin (50 µg/mL) to select for pUC57-Kan or Zeocin (25 µg/mL) to select for pGAPZα A and pPICZα A transformants.

### 3.2. Construction of Expression Plasmids

The synthetic *xynA* gene was amplified without a signal peptide or stop codon from pUC57-Kan containing the optimized codon *xynA* gene by PCR using the following specific primers: xynA-F (5′-GCGGTACCGAGTCATATACTTCTTCCTCAACC-3′), and xynA-R (5′-GAGCGGCCGCGGAAACGGTAATGTCAGCG-3′). *Kpn*I-HF and *Not*I-HF restriction sites (underlined) were added to the forward and reverse primers, respectively. The PCR was thermal cycled at 98 °C for 30 s followed by 30 cycles of 98 °C for 10 s, 54.1 °C for 30 s and 72 °C for 20 s, and then a final 72 °C for 10 min. The amplified fragments were purified and then digested with *Kpn*I-HF/*Not*I-HF before cloning into the pGAPZα A or pPICZα A expression vectors predigested with the same restriction enzymes to yield the pGAPZαA-xynA and pPICZαA-xynA plasmids, respectively. The plasmids were transformed into *E. coli* DH5α competent cells and selected by plating on low-salt LB agar plates (1% (*w/v*) tryptone, 0.5% (*w/v*) yeast extract, 0.5% (*w/v*) NaCl, 1.5% (*w/v*) agar) containing 25 µg/mL Zeocin to select for the presence of the recombinant plasmid and isolate single colonies. The insertion was verified by double restriction enzyme digestion and commercial DNA sequencing (Pacific Science, Bangkok, Thailand).

### 3.3. Yeast Transformation and Selection

The pGAPZαA-xynA and pPICZαA-xynA plasmids were linearized with *Avr*II and *Pme*I, respectively, for integration into *P. pastoris* genome. Each linearized DNA was transformed into *P. pastoris* strain X-33 using the lithium acetate and dithiothreitol method by electroporation [55]. Briefly, about 10 µg linearized plasmid combined with 25 µg salmon sperm DNA (Invitrogen) was used to transform 90 µL competent *P. pastoris* X-33 cells using a Gene Pulser Xcell (Bio-Rad) and immediately recovered in 1 mL of 1 M sorbitol for 2 h at 30 °C. Transformants were selected on YPDS agar plates containing 100–2000 µg/mL Zeocin. Transformants under the control of *P_GAP_* were chosen for preliminary selection on YP agar plates containing 0.2% (*w/v*) Azo-xylan.

### 3.4. Expression of Recombinant xynA-PC in P. pastoris

For constitutive expression (*P_GAP_*), *P. pastoris* transformants were cultured in YPD medium for 4 days, whereas for methanol-inducible expression (*P_AOX1_*), *P. pastoris* transformants were grown using buffered glycerol-complex medium (BMGY: 1% (*w/v*) yeast extract, 2% (*w/v*) peptone, 100 mM KH_2_PO_4_/KOH buffer (pH 6.0), 1.34% (*w/v*) Yeast Nitrogen Base, 4 × 10^−5^% (*w/v*) biotin, and 1% (*v/v*) glycerol) and buffered methanol-complex medium (BMMY: 1% (*w/v*) yeast extract, 2% (*w/v*) peptone, 100 mM KH_2_PO_4_/KOH buffer (pH 6.0), 1.34% (*w/v*) Yeast Nitrogen Base, 4 × 10^−5^% (*w/v*) biotin, and 0.5% (*v/v*) methanol). Absolute methanol was added every 24 h to maintain induction for 5 days, at an initial final concentration of 0.5–1% (*v/v*) in BMMY medium, and then supplemented at 0.5–3% (*v/v*) for the daily induction to optimize recombinant xylanase (Pc Xyn11A) production. All enzyme productions were performed in a baffled shaker flask at 30 °C with constant shaking at 200 rpm. The culture supernatant was collected at periodic intervals to analyze the expression level to determine the optimal time for post-induction harvest. When the maximum xylanase activity was observed, the culture supernatant was collected to analyze the production level and to allow purification and characterization of the produced Pc Xyn11A.

### 3.5. Xylanase Activity Assay and Protein Determination

The xylanase activity was assayed by following the release of reducing sugars from xylan. The reaction mixture consisted of 0.5 mL of 4% (*w/v*) birchwood xylan in deionized water, 0.4 mL of 0.15 M acetate buffer (pH 5.0), and 0.1 mL of suitably diluted enzyme. Reactions were incubated at 50 °C for 5 min and aliquots (75 µL) were taken every minute. The reaction was stopped by transferring it into an equal amount of DNS (3,5-dinitrosalicylic acid) before boiling for 5 min [56]. After cooling, 0.75 mL deionized water was added and the amount of soluble reducing sugars was then measured by monitoring the absorbance at 540 nm with a spectrophotometer. One unit (U) of xylanase activity was defined as the amount of enzyme that liberates 1 µmol of xylose equivalents per min per mL under the assay condition. Protein concentration was estimated using the DC Protein Assay (Bio-Rad) with bovine serum albumin (BSA) as a standard.

### 3.6. Purification of the Pc Xyn11A Enzyme

All purification steps were performed at 4 °C. Yeast cells from liquid cultures were sedimented by centrifugation at 4500× *g* for 10 min. The supernatant was concentrated and exchanged against 20 mM acetate buffer (pH 5.5) by ultrafiltration through regenerated cellulose membrane ultra-centrifugal filters with a 10 kDa molecular weight cut-off (MWCO; Amicon) and loaded onto a HiLoadTM 16/10 Q Sepharose High Performance column (GE Healthcare Life Sciences, Uppsala, Sweden) previously equilibrated with 20 mM acetate buffer (pH 5.5). The adsorbed proteins were eluted with a linear gradient of 0–0.6 M NaCl in the same buffer at a flow rate of 2 mL/min.

### 3.7. SDS-PAGE and Zymography Analysis

The MWCO concentrated culture supernatant (crude Pc Xyn11A preparation) and purified Pc Xyn11A were subjected to sodium dodecyl sulfate polyacrylamide gel electrophoresis (SDS-PAGE) using Mini-Protean^®^ TGX Stain-FreeTM gels to determine the molecular weight and confirm the apparent purity (size homogeneity) of the enzyme. The zymographic analysis for xylanase activity was performed on 10% polyacrylamide gel containing 1% (*w/v*) Azo-xylan as substrate. The electrophoresed gel was soaked in 25% (*v/v*) isopropanol to remove the SDS and renature the protein and then washed four times with 60 mM acetate buffer pH 5.0 for 30 min each, before being incubated in the same buffer at room temperature overnight. The activity bands were observed as clear and colorless areas.

### 3.8. Effect of pH and Temperature on Xylanase Activity and Stability

The effect of pH on the xylanase activity of the purified Pc Xyn11A was determined within the pH range of 3.0–7.0 using xylan as a substrate in citric acid–sodium citrate buffer (pH 3.0–6.0) and sodium phosphate buffer (pH 6.0–7.0) at 50 °C, whilst the optimum temperature of the purified Pc Xyn11A was evaluated at 25 °C (room temperature) to 60 °C using xylan as the substrate at the determined pH optimum.

The effect of pH on the purified Pc Xyn11A xylanase stability was evaluated by measurement of the residual activity after 24 h pre-incubation at 25 °C in 60 mM of the appropriate buffers: citric acid–sodium citrate buffer (pH 3.0–6.0), sodium phosphate buffer (pH 6.0–8.0) and sodium carbonate (pH 9.0–10.0), whilst the thermostability was determined after pre-incubation in citric acid–sodium citrate buffer (pH 5.0) at 40–60 °C. Each data point represents the mean of three independent replicates and the error bars indicate the standard deviation.

### 3.9. Kinetic Parameters

The kinetic parameters (*K*_m_, *k*_cat_ and *V*_max_) of the purified Pc Xyn11A enzyme were determined using birchwood xylan at 0.1–2% (*w/v*) as the substrate in 60 mM citric acid-sodium citrate buffer at pH 5.0, 40 °C for 10 min. The kinetic analysis was then performed using Michaelis–Menten and Lineweaver–Berk plots. Each data represents the mean of three independent experiments.

## 4. Conclusions

This is the first report on the expression of the codon-optimized *xynA* gene from *P. citrinum* FERM P-15944 in *P. pastoris* X-33. The data obtained in the present study showed that *P. pastoris* X-33 is a suitable host for expression and secretion of high levels of Pc Xyn11A. In this study, the codon-optimized *xynA* gene, encoding xylanase A, was successfully cloned and overexpressed in *P. pastoris* using the native *Saccharomyces cerevisiae* α-factor secretion signal under the control of the strong constitutive (*P_GAP_*) or methanol-inducible (*P_AOX1_*) promoters. Both promoters demonstrated efficient expression of the codon optimized *xynA* gene. The total xylanase activity produced with the *P_AOX1_* was 193-fold higher than with the native *P. citrinum* strain. The high level of secretion and the stability over a wide range of pH and at moderate temperatures of the Pc Xyn11A make it potentially useful for a variety of biotechnological applications.

## Figures and Tables

**Table 1:** Xylanase activity of XynA from *P. citrinum* in culture supernatant.

Microorganism	Activity (µmol/mL/min)	Comparison with The Native Strain (fold)	References
*P. citrinum* FERM P-15944 the native stain	3.5	-	[21]
*P. pastoris* GS115 + pPIC3.5	17	5	[21]
*E. coli* BL21(DE3) + pEXP401	16.8	5	[36]
*Y. lipolytica* zeta + JMP62UraTEF_natXynAPc	38	11	[37]
*Y.lipolytica* zeta + JMP62UraTEF_fusXynAPc	180.3	52	[37]
*P. pastoris* X-33 + pGAPZαA-xynA	119.5	34	this study
*P. pastoris* X-33 + pPICZαA-xynA	676	193	this study

**Table 2:** Purification of extracellular Pc Xyn11A enzyme.

Step	Total Activity (U)	Total Protein (mg)	Specific Activity (U/mg)	Purification (fold)	Yield (%)
Culture supernatant	81118	2175.57	37.29	1.00	100
Ultrafiltration (10 kDa MWCO)	77305	533.25	144.97	3.89	95.3
Q Sepharose anion exchange	21425	17.02	1258.96	33.77	26.4

**Table 3:** Comparison of enzymatic properties between Pc Xyn11A and those of other family 11 β-1,4 xylanases.^a^

Enzyme	*K*_m(app)_ (mg/mL)	*k*_cat_ (s^−1^)	*k*_cat_/*K*_m(app)_ (mL/mg/s)	References
Native XylA^b^	6.8	85	12.5	[52]
reXylA^c^	12.6	150	11.9	[52]
Nf Xyn11A^d^	6	136.9	22.8	[53]
re-XynA^e^	5.2	245	47.1	[37]
Pc Xyn11A	2.8	243	86.8	this study

^a^ Kinetic values were analyzed using birchwood xylan as substrate. ^b^ Native *Aspergillus niger* xylanase. ^c^ Recombinant *Aspergillus niger* xylanase produced in *Pichia pastoris.* ^d^ *Nonomuraea flexuosa* xylanase produced in *Trichoderma reesei.* ^e^ *Penicillium citrinum* xylanase produced in *Yarrowia lipolytica*.
